# Relationship between CH_3_OD Abundance and Temperature in the Orion KL Nebula

**DOI:** 10.1021/acs.jpca.2c01309

**Published:** 2022-08-24

**Authors:** Olivia H. Wilkins, Geoffrey A. Blake

**Affiliations:** † Division of Chemistry and Chemical Engineering, California Institute of Technology, Pasadena, California 91125, United States; ‡ Division of Geological and Planetary Sciences, California Institute of Technology, Pasadena, California 91125, United States

## Abstract

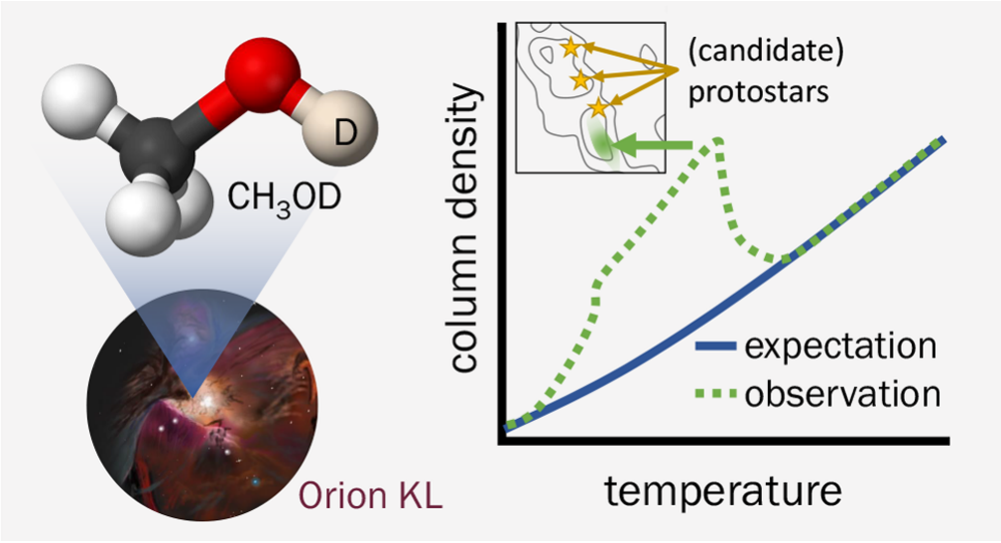

The relative abundances of singly deuterated methanol isotopologues, [CH_2_DOH]/[CH_3_OD], in star-forming regions deviate from the statistically expected ratio of 3. In Orion KL, the nearest high-mass star-forming region to Earth, the singly deuterated methanol ratio is about 1, and the cause for this observation has been explored through theory for nearly three decades. We present high-angular resolution observations of Orion KL using the Atacama Large Millimeter/submillimeter Array to map small-scale changes in CH_3_OD column density across the nebula, which provide a new avenue to examine the deuterium chemistry during star and planet formation. By considering how CH_3_OD column densities vary with temperature, we find evidence of chemical processes that can significantly alter the observed gas-phase column densities. The astronomical data are compared with existing theoretical work and support D–H exchange between CH_3_OH and heavy water (i.e., HDO and D_2_O) at methanol’s hydroxyl site in the icy mantles of dust grains. The enhanced CH_3_OD column densities are localized to the Hot Core-SW region, a pattern that may be linked to the coupled evolution of ice mantle chemistry and star formation in giant molecular clouds. This work provides new perspectives on deuterated methanol chemistry in Orion KL and informs considerations that may guide future theoretical, experimental, and observational work.

## Introduction

The relative abundances of site-specific stable isotopologues, particularly those involving deuterated compounds, are powerful tools that can be used to trace chemical evolution in the interstellar medium and during star and planet formation. For example, the relative abundances of heavy water (i.e., HDO and D_2_O) in comets and meteorites can provide insights into the processing of water between the primordial molecular cloud and present-day Earth.^1,2^ Interstellar compounds such as N_2_H^+^ and CH_3_OH have D/H ratios that are higher than the cosmic value of ∼10^–5^,^3^ and that ratio is a function of temperature, with higher D/H ratios signaling formation in colder, typically denser, environments.^4^

In many high-mass and low-mass protostars alike, the deuterium chemistry of methanol—namely, the relative abundances of the singly deuterated isotopomers CH_2_DOH and CH_3_OD—has presented itself as a mystery. Methanol is one of the simplest complex (having ≥6 atoms) organic molecules, and it is found at every stage of star formation, from cold cloud cores and hot cores/corinos to outflows and circumstellar disks.^5,6^ As such, it is commonly used as a tracer of other complex organics.

In prestellar and protostellar cores, methanol forms primarily via successive hydrogenation of frozen CO on grain mantles (eq 1).^7,8^
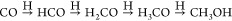
1Statistically, we would expect the [CH_2_DOH]/[CH_3_OD] ratio to be 3 since there are three methyl hydrogen sites compared to a single hydroxyl site. This statistical ratio has been observed in the massive star-forming region NGC 7538-IRS1 but not toward many other star-forming regions.^9^ Low-mass cores generally exhibit ratios >3 and as much as ≳10,^10,11^ while high-mass protostars tend to have [CH_2_DOH]/[CH_3_OD] ratios of <3.^12−14^ Astrochemical models have predicted that the [CH_2_DOH]/[CH_3_OD] ratio should be ≥10 in prestellar cores and that CH_3_OD is only efficiently formed on icy grains at later evolutionary stages when the ices are warmed due to the presence of young (proto)stars.^15^ Deviations from the statistical ratio in high-mass star-forming regions have also been attributed to grain surface chemistry,^16,17^ but investigations into the intricacies of such processes—and the potential role of gas processing—are ongoing.

The Orion Kleinmann-Low (Orion KL) nebula is a high-mass star-forming region notable for its peculiar methanol deuteration. At a distance of ∼388 pc,^18^ Orion KL is uniquely situated to explore the relationship between relative deuterated methanol abundances and environmental conditions because subenvironments within the nebula can be resolved, even with modest imaging capabilities. The two most well-studied regions within Orion KL are the Hot Core and Compact Ridge. The Hot Core region contains denser and warmer gas ( cm^–3^, *T*_kin_ ∼ 200 K) whereas the Compact Ridge, to the southwest,^a^ is cooler and less dense ( cm^–3^, *T*_kin_ ∼ 100–150 K).^19,20^ These regions are also the prominent sites of nitrogen-bearing and oxygen-bearing compounds, respectively.^19,21^ Extending southwest from the Hot Core toward the Compact Ridge is the Hot Core-SW, which is physically and chemically heterogeneous, with possible sources of internal heating^22^ and both oxygen- and nitrogen-bearing compounds.^23,24^ Flanking these regions are compact sources, such as Source I—an edge-on disk thought to be internally heated^25,26^—to the west within the Hot Core region and the (sub)millimeter sources SMA1 and C22—a protostar and possible hot core, respectively^21,27^—to the southwest of Source I and along the northwestern edge of the Hot Core-SW.

Jacq et al.^28^ reported the first definitive detection of CH_2_DOH toward Orion KL on angular scales between 12″ and 26″ (centered on the Hot Core and Source I region). They combined their measurements with past CH_3_OD measurements to report a [CH_2_DOH]/[CH_3_OD] ratio in the range of 1.1–1.5. Neill et al.^29^ similarly reported a ratio of 1.2 ± 0.3 based on local thermodynamic equilibrium models of ∼30″–44″ observations of the Hot Core and the Compact Ridge, while Peng et al.^30^ reported an even lower ratio of 0.7 ± 0.3 toward Orion KL, using an angular resolution of 3.6″ × 2.3″.

There has been extensive debate about whether the apparent CH_3_OD enhancements are the result of grain-surface or gas-phase processes. Early on, Jacq et al.^28^ concluded that their observed ratio was evidence of grain-surface processing followed by injection into the gas phase, perhaps by thermal desorption. Shortly after, chemical models of gas-phase exchange rejected the grain-surface hypothesis on the premise that such chemistry would require unrealistically high [HDO]/[H_2_O] ratios.^31^ Rodgers and Charnley^32^ criticized the assumed statistical [CH_2_DOH]/[CH_3_OD] ratio of 3 since D and H react with species other than CO and H_2_CO, which could affect the relative abundances of the singly deuterated methanol isotopologues. Osamura et al.^33^ used models to suggest that ion–molecule reactions in the gas phase lead to the loss of CH_3_OD, which they conclude accounts for high [CH_2_DOH]/[CH_3_OD] ratios in low-mass star-forming regions, provided methanol is efficiently regenerated in the dissociative recombination of protonated methanol with electrons. That work also finds that D–H exchange on the methyl site is inefficient.

Nevertheless, D–H exchange at the hydroxyl group of methanol on icy grain mantles has emerged as a favored explanation for the [CH_2_DOH]/[CH_3_OD] ratios observed in massive star-forming regions.^17,30,34^ In this mechanism, deuterated water in the ice reacts with CH_3_OH to produce CH_3_OD:
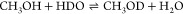
2
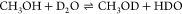
3However, the intricacies of this exchange are still being investigated.

This work provides a new observational perspective on the possibility of D–H exchange at the methanol hydroxyl site by mapping gas-phase CH_3_OD abundances in Orion KL at subarcsecond (∼0.7″) angular resolution using the Atacama Large Millimeter/submillimeter Array (ALMA), which corresponds to linear scales of ∼270 au at the nebula’s distance. This allows us to plot gas-phase CH_3_OD column density as a function of the local line-of-site temperature across relatively small scales within the nebula and explore temperature-dependent chemical processes that may affect the observed CH_3_OD chemistry.

## Methods

### Observations

Observations of Orion KL were taken in ALMA Band 4 during Cycle 5 (project code: ADS/JAO.ALMA#2017.1.01149, PI: Wilkins) on December 14, 2017, completely on the main 12 m array. The pointing center was set to α_J2000_ = 05^h^35^m^14.50^s^, δ_J2000_ = −05°22′30.9″. These observations employed 49 antennas during one execution block. All spectra were obtained in a single local oscillator setup consisting of 10 spectral windows; as such, the uncertainties for quantities derived from these spectra are dominated by thermal noise and mostly unaffected by calibration uncertainty. Of these spectral windows, the targeted CH_3_OD lines were contained in three spectral windows with a spectral resolution of 244 kHz (∼0.5 km s^–1^) covering 143.51–143.97, 153.16–153.40, and 154.84–155.07 GHz. Projected baselines were between 15.1 m and 3.3 km (7.6 and 1650 kλ, where λ ∼ 2 mm is the wavelength), and the primary beam was 39.1″. The on-source integration time was 2062 s. Precipitable water vapor was 3.7 mm, and typical system temperatures were around 75–125 K.

Calibration was completed by using standard CASA (ver. 5.1.1-5) calibration pipeline scripts. The source J0423-0120 was used as a calibrator for amplitude, atmosphere, bandpass, pointing, and WVR (water vapor radiometer) variations, and J0541-0211 was used as the phase and WVR calibrator.

The CH_3_OD data introduced here were prepared in the same way as the ^13^CH_3_OH images presented by Wilkins et al.^22^ In brief, the data cubes were created from measurement sets split to include only baselines of ≤500 m, resulting in a synthesized beam of 0.74″ × 0.63″. Cubes were reduced with continuum emission estimated from line-free channels subtracted by using the uvcontsub function followed by imaging using the tclean algorithm with robust weighting, a Briggs parameter of 1.5 (i.e., seminatural weighting) for deconvolution, and the “auto-multithresh” masking algorithm^35^ in conjunction with interactive tclean. The images have a noise level of σ_RMS_ ∼ 1.3 mJy beam^–1^.

### Deriving CH_3_OD Parameters

The CH_3_OD column density as a function of position was derived via pixel-by-pixel fits of the CH_3_OD transitions shown listed in Table 1 assuming optically thin lines (see Table S1 of the Supporting Information) in local thermodynamic equilibrium (LTE).^b^ Integrated intensity maps of each transition are providing in the Supporting Information (Figure S2). For each coordinate-space pixel in the data cubes, a spectrum within a single synthesized beam centered on that pixel was extracted. The CH_3_OD rotational temperature (*T*_rot_) profile was assumed to be the same as that previously derived^22^ from ^13^CH_3_OH because the transitions of both isotopologues have similar upper energy states *E*_u_. The column density, line width (∼0.8–3.5 km s^–1^), and local standard of rest (LSR) velocity (∼7–9 km s^–1^) were determined by simultaneous fits using LMFIT, a least-squares fitting software package.^c^ Because all lines used in the fit were observed simultaneously, the uncertainties in excitation, which are derived from relative fluxes, should be dominated by thermal noise rather than by multiple sources of calibration uncertainty.

**Table 1:** Transitions of CH_3_OD Used for Line Fits^a^

transition	ν (GHz)	*E*_u_ (K)	*S*_*ij*_μ^2^ (D^2^)	*g*_u_
5_(1,4)_–5_(0,5)_ A	143.7417	39.48	11.2	11
7_(1,6)_–7_(0,7)_ A	153.3240	68.05	14.7	15
3_(−1,2)_–2_(0,1)_ E	154.9628	17.71	2.2	7

a From Anderson et al.^36^ ν = rest frequency of the transitions; *E*_u_ = upper state energy; *S*_*ij*_μ^2^ = product of the transition line strength and the square of the electric dipole moment; *g*_u_ = upper-state degeneracy.

## Results and Discussion

### CH_3_OD Column Density

**Figure 1:**
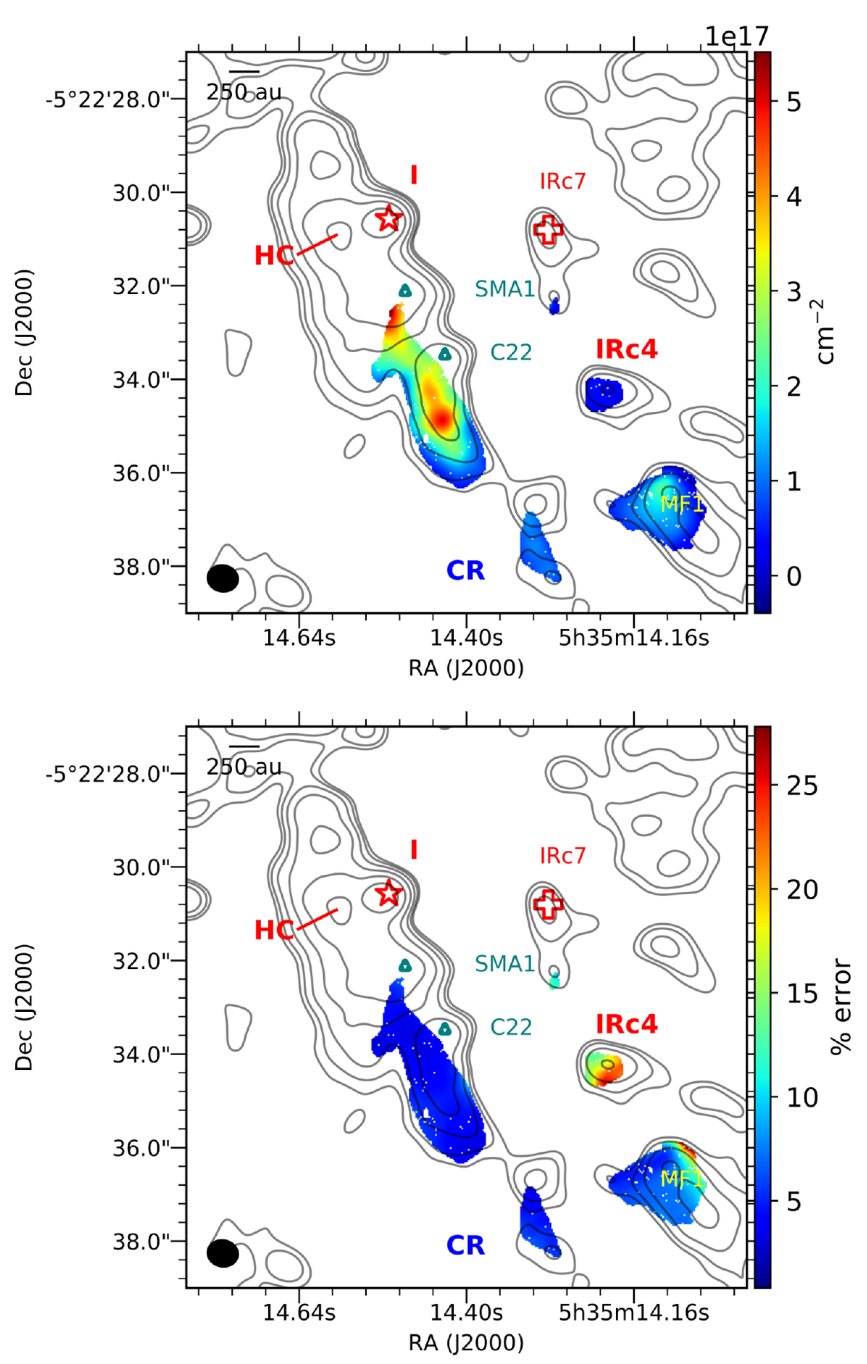
Derived CH_3_OD column density and percent propagated uncertainty shown by the color maps in the upper and lower panels, respectively. The 2 mm (∼150 GHz) continuum emission is shown by the gray contours at 2σ_RMS_, 4σ_RMS_, 8σ_RMS_, 16σ_RMS_, 32σ_RMS_, and 64σ_RMS_. The Hot Core (HC), Source I (I), IRc7, and IRc4 are shown in red; SMA1 and C22 are shown by the teal diamonds; the methyl formate emission peak (MF1)^24^ is labeled in yellow, and the Compact Ridge (CR) is labeled in blue. The 0.7″ synthesized beam is shown by the black ellipse in the bottom left corner.

**Figure 2:**
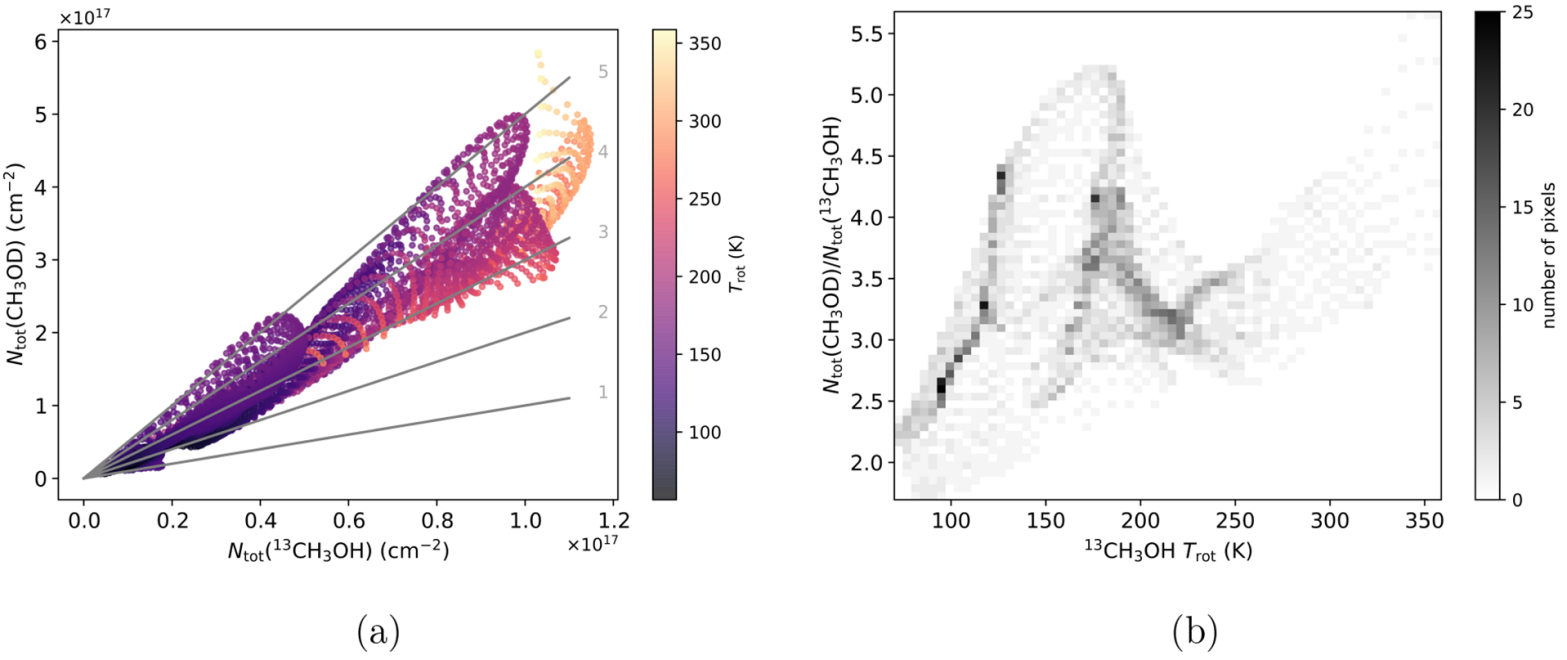
(a) Column densities *N*_tot_ of ^13^CH_3_OH (horizontal axis, from Wilkins et al.^22^) and CH_3_OD (vertical axis, this work) with each point representing a single pixel in Figure 1. Each pixel is colored by its rotational temperature. The gray lines are labeled by the [CH_3_OD]/[^13^CH_3_OH] ratios (1 to 5) they represent. (b) Two-dimensional histogram (50 points per bin) showing the methanol [CH_3_OD]/[^13^CH_3_OH] ratios plotted as a function of temperature.

The column density profile derived from a pixel-by-pixel fit of the ALMA data image cubes was used to show small-scale variations in CH_3_OD column densities across Orion KL. As shown in Figure 1, the derived CH_3_OD column density (*N*_tot_) is generally on the order of 10^17^ cm^–2^ and peaks south of SMA1 and C22. In general, the uncertainties (standard errors calculated by using LMFIT) for these values are <10% throughout the region southwest of the Hot Core (Hot Core-SW), which is the region of interest for most of the discussion in this work; higher uncertainties, up to ∼25%, characterize IRc4 and the western (right) edge of the Compact Ridge. The relationship between the CH_3_OD and ^13^CH_3_OH column densities (Figure S3) and rotational temperature (*T*_rot_, Figure S4) is illustrated by Figures 2a and 2b. In general, [CH_3_OD]/[^13^CH_3_OH] falls between 1.5 and 5.3. Assuming a local interstellar ^12^C/^13^C ratio of 68 ± 15,^37^ this suggests [CH_3_OD]/[CH_3_OH] ≈ 0.015–0.104, which encompasses the ratio of 0.01–0.06 in Orion KL reported by Mauersberger et al.^38^ on much larger angular scales of 15″–23″. The discrepancy on the higher end of these ranges may be the result of CH_3_OD abundance enhancements on smaller spatial scales being diluted in the single-dish data, but otherwise, the agreement suggests that the flux recovered in our observations is representative of that collected by single-dish observations. A comparison of [CH_3_OD]/[^12^CH_3_OH] ratios derived from different assumed ^12^C/^13^C values is presented in Table S2.

**Figure 3:**
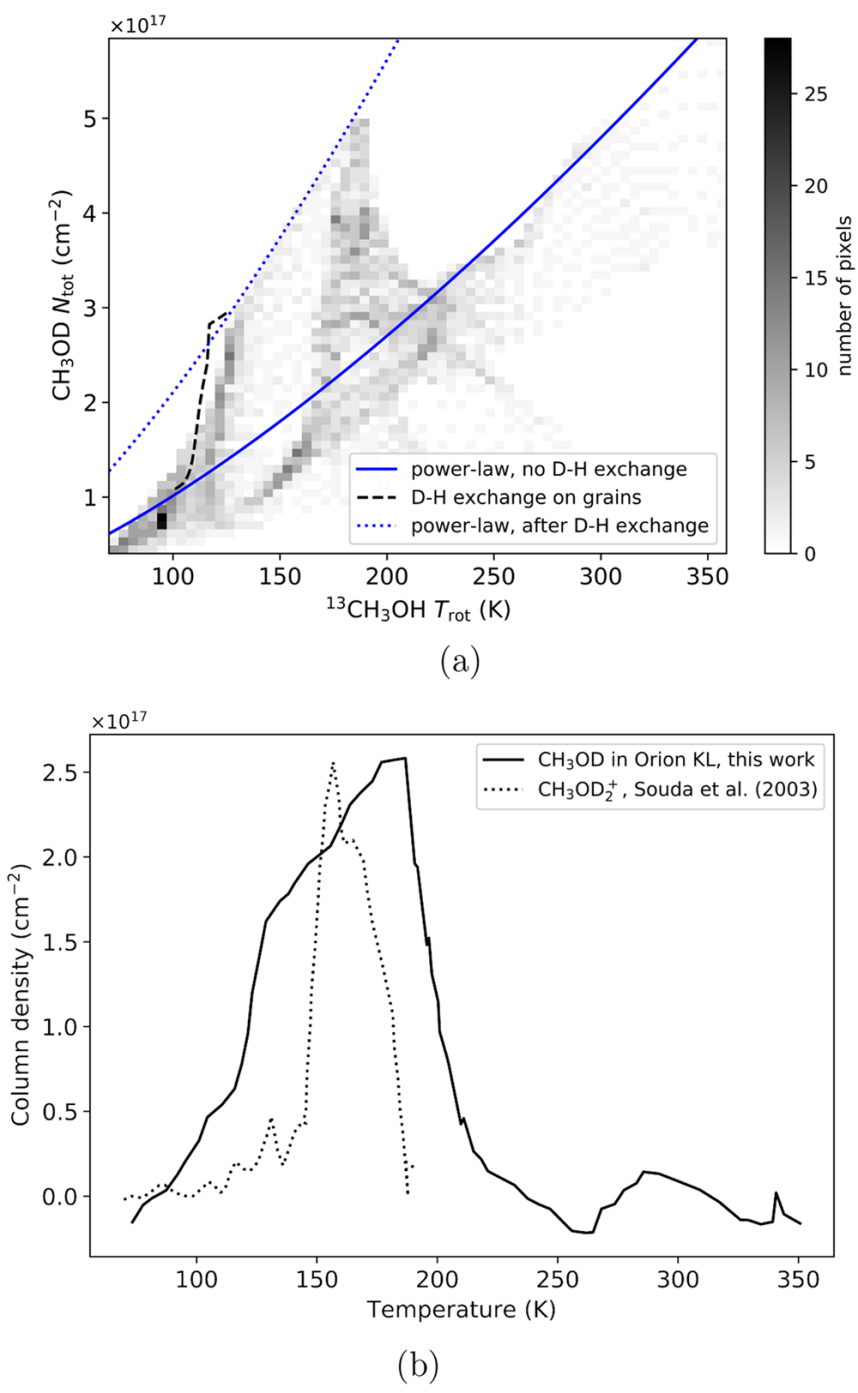
(a) Two-dimensional histogram (50 points per bin) showing the derived CH_3_OD column densities *N*_tot_ against rotational temperature *T*_rot_ of ^13^CH_3_OH. The blue solid line shows the power-law fit to the data if there were no D–H exchange (eq 4). The black dashed line shows the modeled D–H exchange (eq 5) followed by desorption based on ice experiments by Souda et al.^39^ and assumptions by Faure et al.^40^ The blue dotted curve is the power-law fit after the CH_3_OD enhanced by D–H exchange thermally desorbs off the grains. (b) The solid curve shows the density profile with the underlying power-law (blue curve in (a), eq 4) subtracted. The dotted curve shows the sputtered CH_3_OD_2_^+^ profile reported by Souda et al.^39^ but scaled for comparison to the CH_3_OD column density profile in this work.

Figure 3a, in which each point represents 50 binned pixels for which a column density and rotational temperature pair were derived, shows that the CH_3_OD column density increases with rotational temperature, which is characteristic of thermal desorption in which material is sublimed from the grains as the environment warms.^41^ However, the profile also contains a “shark-tooth” feature where the column density starts to rise more steeply at ∼110 K before peaking close to 185 K. At temperatures higher than 185 K, there is a sharp decrease in the CH_3_OD column density, and the relationship between column density and rotational temperature returns to the underlying trend.

Power-law distributions are commonly used to characterize the temperature and density profiles of star-forming regions and young stellar objects (YSOs).^42,43^ In Figure 3a, the fitted underlying power-law relationship between *T*_rot_ and *N*_tot_, described by eq 4, is shown by the solid blue line.
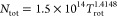
4The solid black line in Figure 3b shows the “shark tooth” from Figure 3a with the underlying power-law between *T*_rot_ and *N*_tot_ subtracted.

### Grain-Surface Processes

The rapid rise in gas-phase CH_3_OD column density between ∼110 and ∼120 K is consistent with D–H exchange between methanol and heavy water (HDO, D_2_O) on the ices at ∼100 K. Souda et al.^39^ experimentally investigated hydrogen bonding between water and methanol in low-temperature ices warmed from 15 to 200 K under ultrahigh-vacuum conditions. They observed that when CH_3_OH was adsorbed onto D_2_O ice, secondary CH_3_OD_2_^+^ ions—evidence of D–H exchange at the hydroxyl site—sputtered off the ice analogue surfaces predominantly between 140 and 175 K. Follow-up analyses by Kawanowa et al.^44^ describe this as a “rapid and almost complete H/D exchange” to yield the sputtered CH_3_OD_2_^+^ species. The fact that we see a similar sudden increase in CH_3_OD column densities at similar temperatures (Figure 3b, dotted line), with discrepancies due to the differences in pressure between ultrahigh vacuum and even the densest regions of interstellar medium, supports a similar rapid exchange in Orion KL.

Models of D–H exchange between water and methanol in ices by Faure et al.^40^ successfully reproduced gas-phase CH_3_OH deuterium fractionation in Orion KL using initial ice abundances of *n*_S_(CH_3_OH) = 2.0 × 10^–6^*n*_H_, *n*_S_(HDO) = 3.0 × 10^–7^*n*_H_, and *n*_S_(CH_3_OD) = 6.0 × 10^–9^*n*_H_. Taking these initial ice abundances, we modeled the change in gas-phase CH_3_OD column density following rapid D–H exchange on the ices and subsequent desorption. Specifically, we assumed an initial ice column density of *N*_S_(CH_3_OD) = 6.0 × 10^–9^*N*_H_ = 6.0 × 10^14^ cm^–2^, since *N*_H_ ∼ 10^23^ cm^–2^ across Orion KL (including the Hot Core, Compact Ridge, and Extended Ridge),^45,46^ and an initial gas-phase column density of *N*(CH_3_OD) = 1 × 10^16^ cm^–2^, based on the column densities measured at 100 K in this work after subtracting the underlying power law in eq 4.

The enhancement of gas-phase CH_3_OD column density from D–H exchange with water was then modeled. In the absence of directly analogous temperature-programmed desorption measurements of CH_3_OH and CH_3_OD themselves, we fit the CH_3_OD_2_^+^ curve of Souda et al.^39^ (Figure 3b, dotted line) between 110 and 145 K. The relative intensity amplitude was normalized to the column densities observed for CH_3_OD in our Orion KL observations, resulting in a relationship of the form
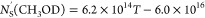
5where *N*_S_^′^ is the additional (solid) CH_3_OD available [cm^–2^] for desorption at a given temperature *T*. The desorption rate coefficient is expressed as
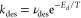
6where the pre-exponential factor ν_des_ and the binding energy *E*_d_ are taken to be approximately the values for annealed amorphous solid water—2.0 × 10^12^ s^–1^ and 5200 K, respectively—under the assumption that the methanol desorbs with water, which is in excess.^40,47−51^

It follows that the change in gas-phase *N*(CH_3_OD) is approximated by multiplying the rate coefficient (eq 6) by the total solid CH_3_OD column density, which includes the additional solid CH_3_OD available following D–H exchange (eq 5) at temperature *T*. Thus, the rate at which *N*(CH_3_OD) changes in the gas phase between 100 and 150 K is approximated by
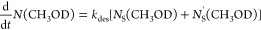
7Using temperature steps of 1 K, we determined the corresponding time steps by
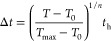
8where Δ*t* is the time elapsed since *t* = 0; *T*_0_ and *T*_max_ are the initial (10 K) and maximum (300 K) temperatures, respectively; *t*_h_ is the heating time scale; and *n* is the order of heating, which is assumed to be 2 following the previous work.^52^ The initial gas and solid CH_3_OD column densities were assumed respectively to be *N*(CH_3_OD) = 9.0 × 10^15^ cm^–2^ (approximated from the CH_3_OD density profile with the underlying power-law subtracted) and *N*_S_(CH_3_OD) = 6.0 × 10^–9^*N*_H_ cm^–2^ (based on assumptions used by Faure et al.^40^ in their D–H exchange models).

The resulting desorption model from eq 7 with *t*_h_ = 10^3^ years is shown by the black dashed line in Figure 3a. Longer time scales (i.e., *t*_h_ ≥ 10^4^ years) characteristic of massive YSOs do not follow the increasing CH_3_OD profile as closely. Although potential internal heating sources have been suggested within the Hot Core-SW,^22^ Li et al.^53^ conclude that this region, part of the “elongated ridge” comprising the Hot Core and Source I, is predominantly heated externally by shocks induced by the Orion KL explosion, which took place about 500 years ago. Furthermore, the models by Faure et al.^40^ suggest the D–H exchange in Orion KL reaches steady state in <10^3^ years. After D–H exchange, the power-law relationship from eq 4 applies, but with a coefficient of 3.2 × 10^14^ (dotted blue curve in Figure 3a).

The hypothesis that the CH_3_OD column density profile in Figure 3 is the result of rapid D–H exchange on the grains relies on two assumptions regarding temperature. First, we assume that the rotational temperature *T*_rot_ is an appropriate estimate for the kinetic temperature *T*_kin_. This assumption is based on the fact that the Compact Ridge, a spatial component toward the southwestern region of Orion KL that is characterized as being rich in oxygen-bearing molecules, has a fairly high density of ∼10^6^ cm^–3^,^19,20^ implying that LTE is a reasonable assumption. Second, we assume that the dust and gas are thermally coupled. Li et al.^54^ found that other quiescent regions (no infrared sources, no evident outflows) in the Orion Molecular Cloud are thermally coupled. Models by Bruderer et al.^55^ also support coupling between dust and gas temperature at the relevant densities. This gas-grain thermal coupling was also demonstrated in models of several massive star-forming regions, including the Orion KL Compact Ridge, by Garrod and Herbst.^56^ As such, it is reasonable to assume that the gas temperatures shown in Figure 3 are also representative of the temperatures of the dust, on which methanol forms, and that any decoupling between the dust and gas is negligible for the purposes of this discussion.

### Enhanced Deuteration in the Hot Core-SW

**Figure 4:**
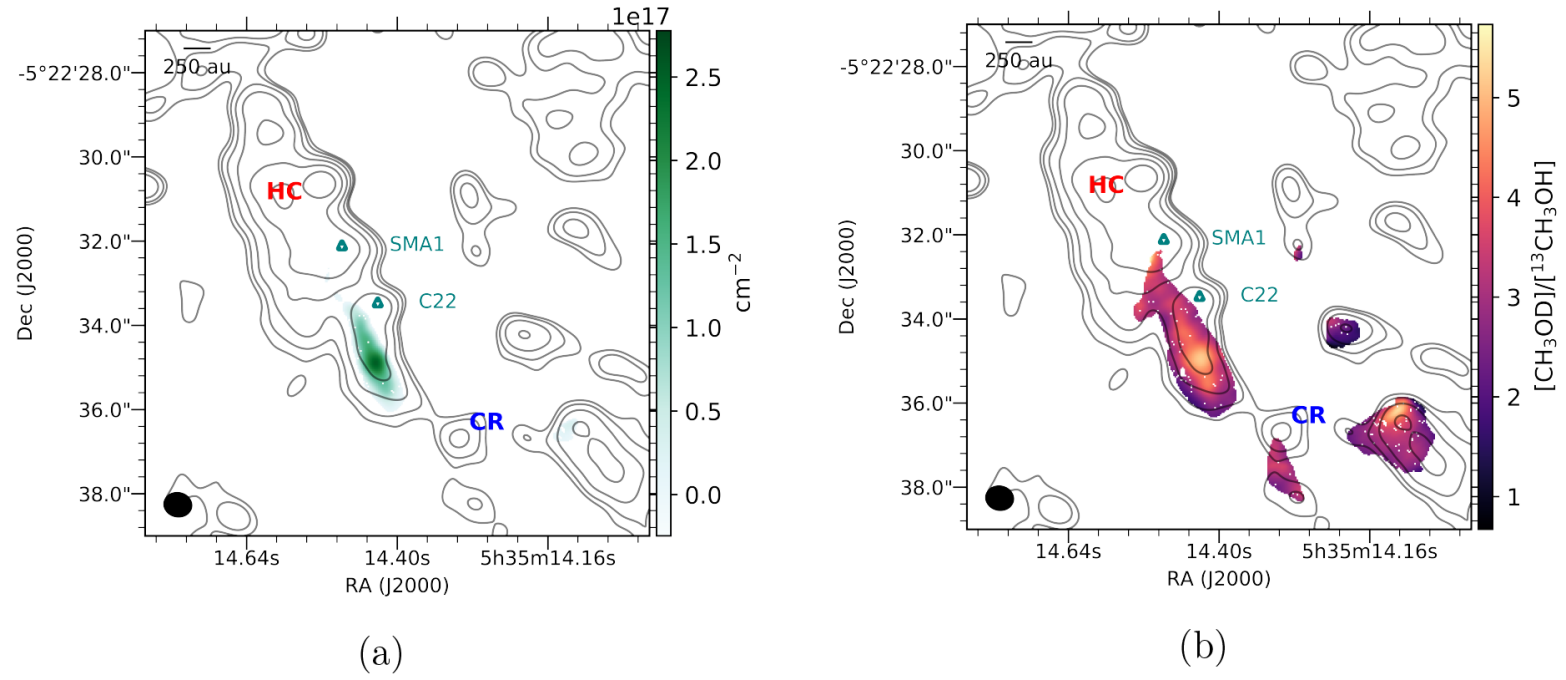
(a) Map of gas-phase CH_3_OD excess column density across Orion KL after subtracting the underlying power law relationship (eq 4) between rotational temperature and column density. Darker shading indicates a larger deviation from the underlying power law. (b) Map of [CH_3_OD]/[^13^CH_3_OH].

Above ∼180 K, the profile in Figure 3a drops sharply. We attribute this to the enhanced deuteration being localized to the Hot Core-SW and that there simply is little gas above 180 K in this region. Figure 4a shows the distribution of CH_3_OD column densities minus the underlying power law relationship (eq 4). Figure 4b maps [CH_3_OD]/[^13^CH_3_OH], confirming that the profile in Figure 4a is indeed the result of excess CH_3_OD in the Hot Core-SW and not enhanced abundances of methanol in general.

We considered two chemical explanations for this trend, neither of which adequately explain the observed patterns. One avenue that has been proposed for CH_3_OD depletion is gas-phase D–H exchange via protonation of the hydroxyl group (eqs 9 and 10) followed by dissociative recombination (eqs 11 and 12).^33^
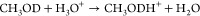
9
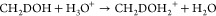
10
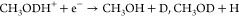
11
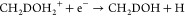
12The methyl H/D site is not exchangeable; therefore in this model, only CH_3_OD can be depleted while CH_2_DOH cannot, which has been suggested as an explanation for the low relative CH_3_OD abundances in low-mass star-forming regions.^33^ However, this is unlikely to account for the decrease in gas-phase CH_3_OD at warmer temperatures in the Orion KL Hot Core-SW following enrichment on grain surfaces because dissociative recombination reactions tend to destroy gas-phase methanol (and its isotopologues), with methanol production comprising the smallest branching ratio (3%) listed in KIDA.^57^ A drop in the ^13^CH_3_OH column density at temperatures above 180 K is not seen in Orion KL (see Figure S5), suggesting that the observed decrease of the [CH_3_OD]/[^13^CH_3_OH] ratio (Figure 2b) and of the CH_3_OD column density (Figure 3a) above 180 K cannot simply be explained by protonation of the methanol (e.g., by H_3_O^+^) followed by dissociative recombination.

Another mechanism considered was the neutral-radical reaction with the hydroxyl radical (OH); however, this reaction is too slow at ∼200 K to account for the observed patterns (see Section S4 of the Supporting Information).

Instead, we propose that the drop in enhanced CH_3_OD column density is the result of environment rather than chemistry. That is the enhanced CH_3_OD column density profile is limited to warm gas in the Hot Core-SW with temperatures that max out around 200 K. The enhanced deuteration in this region, brought about by temperature-dependent surface D–H exchange, may be the result of evolutionary state. However, this region is not directly associated with any known YSOs, including SMA1 (a young, high-mass protostar) and C22 (a possible hot core).^21,27^ Although there is no known self-illuminated source (e.g., embedded protostar) in the Hot Core-SW to drive grain warming and associated D–H exchange in ices, it has been suggested that there is a potential (hidden) source of internal heating there.^22^ And, the complex history and star formation patterns in Orion KL will lead to quite varied thermal histories of various subregions of the giant molecular cloud complex. Thus, dedicated work to elucidate the nature of the Hot Core-SW region is needed to better understand how this environment could affect the observed chemistry.

### Methyl Group Chemistry

**Figure 5:**
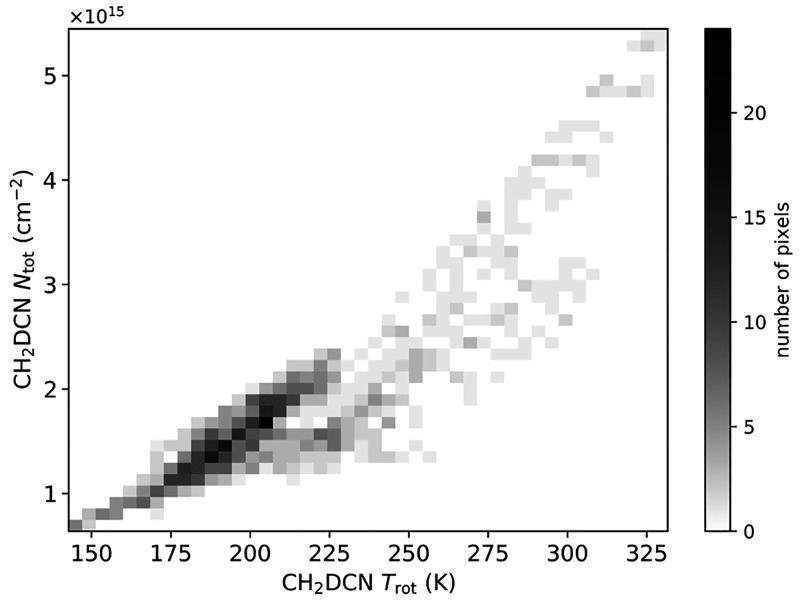
Two-dimensional histogram with 20 points per bin showing the derived CH_2_DCN column density against rotational temperature using data presented by Carroll.^58^

If the hump observed in the CH_3_OD column density versus temperature profile is indeed evidence of surface D–H exchange at the hydroxyl site, then we would expect a smooth profile (i.e., without a similar hump) in the profile of CH_2_DOH. Unfortunately, we do not have sufficient CH_2_DOH transitions in our data to test this hypothesis directly. However, Carroll^58^ mapped the physical parameters of CH_2_DCN toward Orion KL using data from ALMA (project: ADS/JAO.ALMA#2013.1.01034, PI: Crockett). Figure 5 shows a histogram of the CH_2_DCN column density and rotational temperature using these data where they overlap with CH_3_OD emission in the current observations. In this plot, we see a consistent power-law relationship between temperature and column density, which supports the conclusion by Osamura et al.^33^ that the methyl groups of complex organics would not undergo grain-surface or gas-phase D–H exchange. In other words, the hump visible in Figure 3a is indeed likely the result of chemistry specific to the hydroxyl site of methanol. Future dedicated observations of CH_2_DOH lines with ALMA in this region are necessary to confirm this hypothesis.

### Comparison to Past Studies

The evidence presented here adds to the growing list of observational and theoretical evidence in favor of a grain-surface mechanism for CH_3_OD enrichment in massive star-forming regions. A key difference between this work and that of past observations is that here we map CH_3_OD column density across much of Orion KL, including the Compact Ridge, whereas past work derives one value for the Compact Ridge as a whole. Furthermore, resolving the small-scale structure of CH_3_OD column densities (and temperature) allows us to look at how column density is related to the line-of-site temperature, something that has not yet been extensively investigated through observations but is now possible with the sensitivity and spatial grasp of ALMA.

Computational models to assess D–H exchange generally have investigated *temporal* variations in relative CH_3_OD abundances at a single temperature or have looked at singly deuterated methanol chemistry across a range of temperatures but less than 140 K.^14,33,40^ Previous models of grain-surface D–H exchange in Orion KL have assumed methanol is completely sublimated at temperatures >110 K, whereas our observations suggest D–H exchange on the ice may be important up to 125 K. Thus, the observations presented here probe a temperature regime beyond that of existing astrochemical models and call for revised models to investigate D–H chemistry at higher temperatures. Specifically, temperature-dependent models of grain-surface D–H exchange in which methanol sublimates completely at higher temperatures (e.g., 125 K) are needed to more robustly explain the patterns observed in this work.

Perhaps the leading criticism of proposed grain-surface chemistry prompting the enhancement of CH_3_OD abundances relative to CH_2_DOH is that such processes would require a large initial [HDO]/[H_2_O] ratio. For example, models by Charnley et al.^31^ suggest that the initial [HDO]/[H_2_O] ratio in the ice mantles would need to be ∼0.1,^31^ which is significantly larger than the ratio of ∼0.003 reported by Neill et al.^45^ for compact regions of Orion KL. However, Thi et al.^59^ suggest that the [HDO]/[H_2_O] ratio can exceed 0.01 in dense (≥10^6^ cm^–3^), warm (*T* > 100 K) regions (such as those observed here) via neutral–neutral reactions—such as the formation of HDO from OH + HD, OD + H_2_, and OD + OH—which may be promising for the hypothesis of a grain-surface CH_3_OD enhancement. Even more promising is a model presented by Faure et al.,^40^ who reproduced observed [CH_2_DOH]/[CH_3_OD] ratios in the Compact Ridge assuming a primitive [HDO]/[H_2_O] fractionation of 0.006, only a factor of 2 larger than the observed ratio reported by Neill et al.^45^ That is, the deuteration states of methanol and water in the icy grain mantles, which are exceedingly difficult to measure directly with previous observational capabilities, are the key initial conditions for modeling this chemistry. JWST will offer greatly improved capabilities to attempt such measurements, going forward.

A remaining question, then, is what makes methanol deuteration in low-mass star-forming regions so different from that in high-mass star-forming regions? Ratajczak et al.^12^ suggest observational biases, namely that because high-mass objects tend to be further away than those low-mass objects where deuterium chemistry has been studied, measurements of the [CH_2_DOH]/[CH_3_OD] ratio may be affected, particularly if the spatial distributions of the two isotopomers are different. High angular resolution mapping of high-mass star-forming regions, like that presented in Figure 1, would address this by comparing the CH_2_DOH and CH_3_OD column densities only where their emission overlapped, as was done for different spectral components of the high-mass star-forming region NGC 6334I by Bøgelund et al.^14^ As stated previously, the observations presented here do not have sufficient CH_2_DOH lines available to test the spatial correlation of site-specific deuterated isotopologues, and dedicated high angular resolution observations targeting low-energy CH_2_DOH lines are necessary to further address this issue.

Another conjecture for the different deuterium fractionation patterns in massive YSOs compared to low-mass star-forming regions is that there is simply less deuteration in massive protostars because of the warmer environments.^14^ Faure et al.^40^ reproduced relative singly deuterated methanol abundances for Orion KL (a high-mass source) and IRAS 16293–2422 (a low-mass object) using kinetic models that were identical except for the initial deuterium fractionation ratios. They reported that the Orion KL Compact Ridge’s gas-phase deuterium chemistry could be modeled assuming similar primitive deuteration of water and methanol ices (∼0.2–0.3%), whereas IRAS 16293’s gas-phase deuterated methanol chemistry required a significantly higher deuterium fractionation in methanol (12%) than water (1%). Their model shows complete methanol desorption by ∼110 K. Such conditions of extreme deuteration only occur in very cold, dense environments where extensive molecular depletion occurs, including that of CO and N_2_. Under such conditions, D_3_^+^ becomes the dominant molecular ion, whose dissociative recombination results in the arrival of hydrogen atoms onto grain mantles with a D/H ratio of >1.

As seen in Figure 3a, the desorption model based on work by Faure et al.^40^ (dashed line) matches nicely the CH_3_OD column density rise between 100 and 110 K; however, the CH_3_OD column density in our data increases at temperatures up to ∼125 K. This slight discrepancy might be addressed by temperature-programmed desorption experiments, for example, studying the release of CH_3_OD directly rather than via sputtered CH_3_OD_2_^+^ detected by Souda et al.^39^ Furthermore, thermal desorption strongly depends on the composition of the underlying surface. Such questions require more robust chemical networks for deuterium chemistry as well as a better understanding of the initial chemical conditions of both high-mass and low-mass star-forming regions.

## Conclusion

We provide observational evidence in support of rapid D–H exchange in methanol-containing ices, specifically at the hydroxyl site, between ∼100 and 125 K in Orion KL, using high angular resolution ALMA Band 4 observations of CH_3_OD to map the small-scale variations in CH_3_OD column density for the first time and to compare the observed column densities to the line-of-site rotational temperatures mapped at the same angular resolution (and derived previously from ^13^CH_3_OH).^22^

We fit power-law relationships and toy models of D–H exchange at methanol’s hydroxyl (−OH) site followed by CH_3_OD thermal desorption to the observed CH_3_OD column density profile in Orion KL. These analyses suggest that D–H exchange and rapid CH_3_OD desorption increase the gas-phase CH_3_OD column density between 100 and 125 K. Enhanced CH_3_OD column densities are limited to the Hot Core-SW, which has been previously suggested to harbor a potential source of internal heating, which could explain the enhanced CH_3_OD column densities between 125 and 185 K. In this interpretation, there is simply little gas in this region at higher temperatures that would display CH_3_OD enhancements.

Future investigations—through observations, experiments, and computational models—are needed to further constrain the peculiar D-methanol chemistry in Orion KL and other star-forming regions. The work presented here would be aided by dedicated high-resolution observations of CH_2_DOH, spectroscopic experiments measuring the kinetics of CH_3_OD formation via D–H exchange in heavy water ice (HDO and D_2_O) and subsequent desorption, and temperature-dependent astrochemical models of possible CH_3_OD loss at higher temperatures (185–225 K).

## Supporting Information Available

Explanation of optical depth approximations (Section S1); supplemental maps (Section S2), specifically CH_3_OD integrated intensity (Figure S2), ^13^CH_3_OH column density (Figure S3), and ^13^CH_3_OH rotational temperature (Figure S4) maps; a comparison of derived [CH_3_OD]/[^12^CH_3_OH] ratios (Section S3); and loss calculations of CH_3_OD by gas-phase reactions with OH (Section S4) (PDF)

## Footnotes

a In astronomical maps, north is up, east is left, and west is right.

b Python script available at https://github.com/oliviaharperwilkins/LTE-fit.

c 10.5281/zenodo.11813.
