# Supplementary material for: Relationship between CH3OD Abundance and Temperature in the Orion KL Nebula
Source: J Phys Chem A. 2022 Aug 24;126(37):6473–82. doi: 10.1021/acs.jpca.2c01309 (PMC9514801; doi:10.1021/acs.jpca.2c01309)
Supplement: Supplementary file 1 — jp2c01309_si_001.pdf [file jp2c01309_si_001.pdf]

Supporting Information: Relationship between CH_3OD Abundance and Temperature in the Orion KL Nebula

Olivia H. Wilkins*,†,§ and Geoffrey A. Blake‡,¶

†*Division of Chemistry and Chemical Engineering, California Institute of Technology, Pasadena, CA 91125, United States*

‡*Division of Chemistry and Chemical Engineering, California Institute of Technology, Pasadena, California 91125, United States*

¶*Division of Geological and Planetary Sciences, California Institute of Technology, Pasadena, California 91125, United States*

§*Present address: NASA Postdoctoral Program Fellow, NASA Goddard Space Flight Center, Greenbelt, Maryland 20771, United States*

E-mail: olivia.h.wilkins@outlook.com

S1 Optical Depth Approximations

The optical depth was calculated as

$$\tau = \frac{g_u}{\Delta V}\frac{N_{\rm tot}}{Q(T_{\rm rot})}\frac{A_{ul}c^3}{8\pi\nu^3}\left(e^{h\nu/kT_{\rm rot}}-1\right)$$

where g_u is the degeneracy in the upper state, Q is the partition function, A_{ul} is the Einstein A coefficient, ν is the frequency of the transition, h is the Planck constant, and k is the Boltzmann constant.[S1] The line parameters are taken from the Splatalogue Database for Molecular Spectroscopy, except for the A_{ul} values for CH_3OD, which are taken to be the values for the same transitions (i.e., same quantum numbers) of CH_3OH in the absence of

published values for CH_3OD. The $3_{(-1,2)}-2_{(0,1)}$ transition of CH_3OD is not listed in Splatalogue and is thus excluded from Table S1. The derived line width ΔV, total column density $N_{\rm tot}$, and rotational temperature $T_{\rm rot}$ are taken from a representative pixel (corresponding to $\alpha_{\rm J2000} = 05^{\rm h}35^{\rm m}14\overset{\rm s}{.}11$, $\delta_{\rm J2000} = -05°22'36\overset{''}{.}4$) with strong emission in the Compact Ridge. These values, along with the associated optical depth approximations, are given in Table S1. An example of the fitted CH_3OD spectra is shown in Figure S1.

Table S1: Optical depth calculation for Compact Ridge pixel.

**$^{13}CH_3OH$**		**CH_3OD**	
ΔV	1.56 km s^{-1}	ΔV	1.46 km s^{-1}
$N_{\rm tot}$	4.48×10^{16} cm^{-3}	$N_{\rm tot}$	1.74×10^{17} cm^{-3}
$T_{\rm rot}$	156.65 K	$T_{\rm rot}$	156.65 K
Transition	τ	Transition	τ
$8_{(0,8)} - 8_{(-1,8)}$	0.23	$5_{(1,4)} - 5_{(0,5)}$ A	0.32
$6_{(0,6)} - 6_{(-1,6)}$	0.24	$7_{(1,6)} - 7_{(0,7)}$ A	0.32
$5_{(0,5)} - 5_{(-1,5)}$	0.22		

S2 Supplemental Maps

Integrated intensity maps of the CH_3OD transitions used in these analyses are shown in Figure S2.

The $^{13}CH_3OH$ column density and rotational temperature profiles used in this manuscript were previously published by Wilkins et al.[S2] The column density and rotational temperature maps are reproduced here as Figures S3 and S4, respectively, for easy comparison to the CH_3OD column density maps presented in this work. Figure S5 shows a two-dimensional histogram of these data.

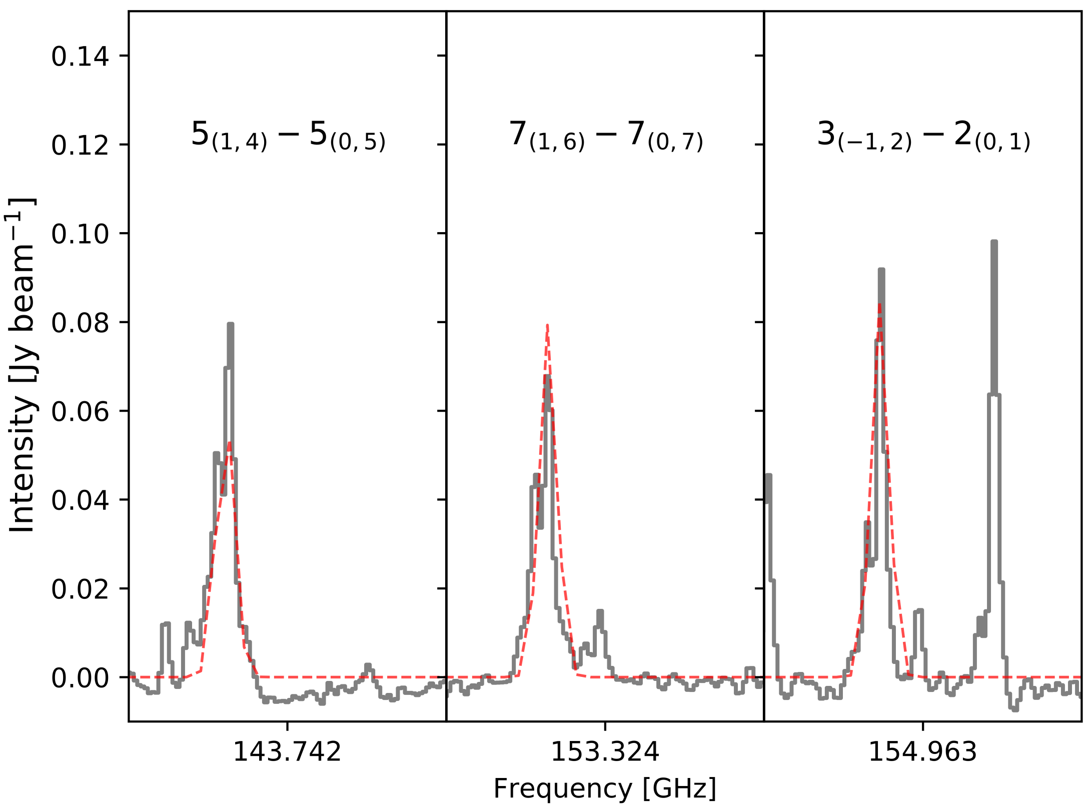

Figure S1: The fitted CH_3OD spectra extracted from a single ALMA beam centered on $\alpha_{J2000} = 05^h35^m14\overset{s}{.}11$, $\delta_{J2000} = -05°22'36\overset{''}{.}4$. Observational data are shown by the solid gray lines with the line fits shown by the dashed red curves.

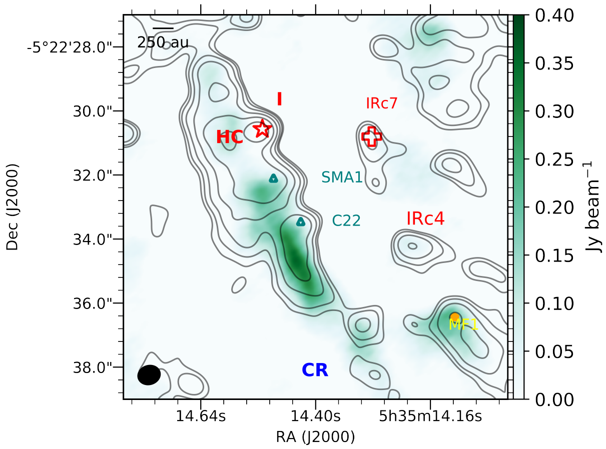

(a) $5_{(1,4)} - 5_{(0,5)}$ A

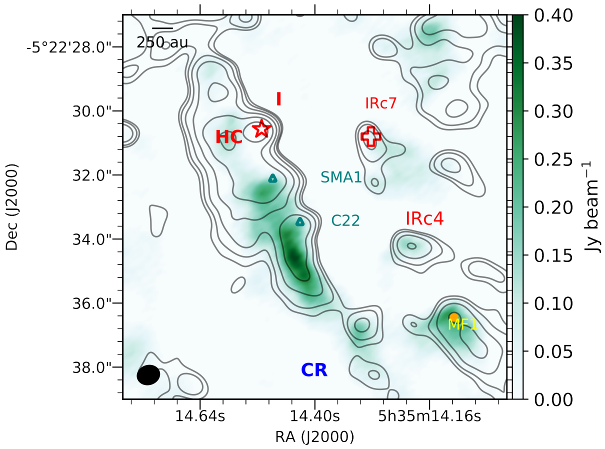

(b) $7_{(1,6)} - 7_{(0,7)}$ A

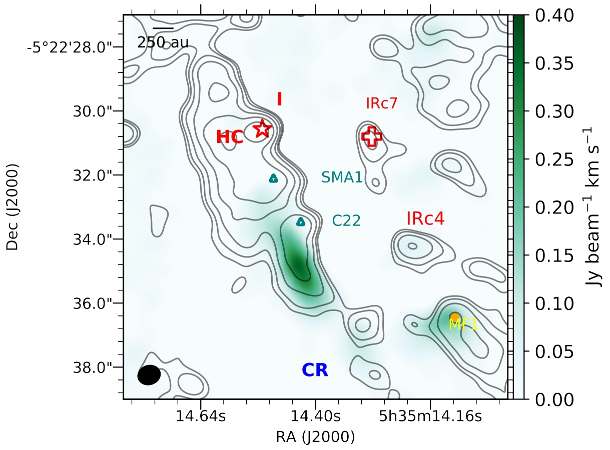

(c) $3_{(-1,2)} - 2_{(0,1)}$ E

Figure S2: Integrated intensity maps of the transitions used to derive the CH_3OD column density in green. The 2 mm (∼150 GHz) continuum emission is shown by the gray contours at $2\sigma_{RMS}, 4\sigma_{RMS}, 8\sigma_{RMS}, 16\sigma_{RMS}, 32\sigma_{RMS}, 64\sigma_{RMS}$. The Hot Core (HC), Source I (I), IRc7, and IRc4 are shown in red; SMA1 and C22 are shown by the teal diamonds; the methyl formate emission peak (MF1)[S3] is labeled in yellow; and the Compact Ridge (CR) is labeled in blue. The orange marker near MF1 indicates the representative pixel chosen for the optical depth calculation in Section S1. The 0.″7 synthesized beam is shown by the black ellipse in the bottom left corner.

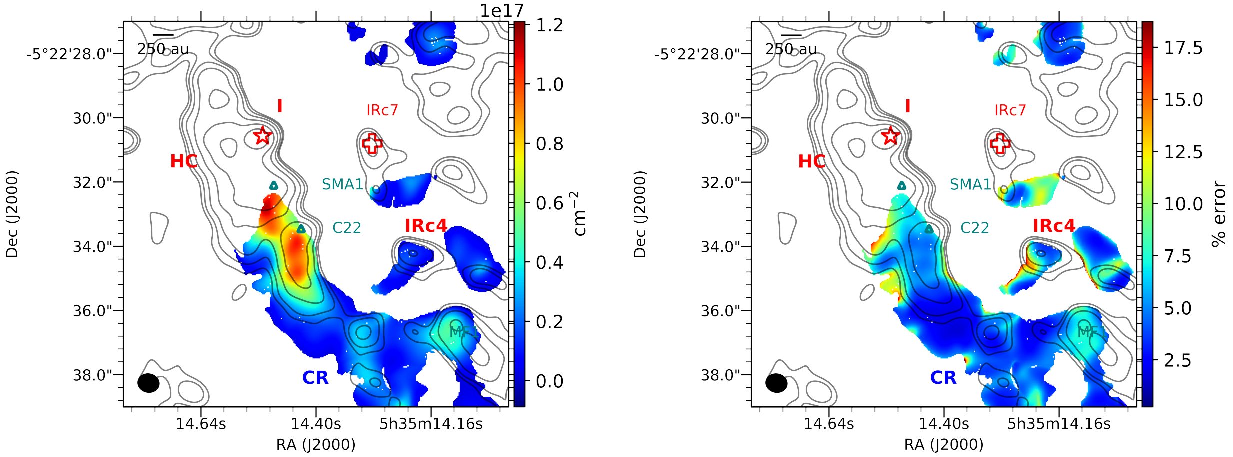

Figure S3: Derived $^{13}CH_3OH$ column density and percent propagated uncertainty derived by Wilkins et al.[S2] shown by the left and right color maps, respectively. The 2 mm (∼150 GHz) continuum emission is shown by the gray contours at $2\sigma_{RMS}, 4\sigma_{RMS}, 8\sigma_{RMS}, 16\sigma_{RMS}, \ldots$ The $0\farcs7$ synthesized beam is shown by the black ellipse in the bottom left corner. Reproduced with permission from Ref. S2.

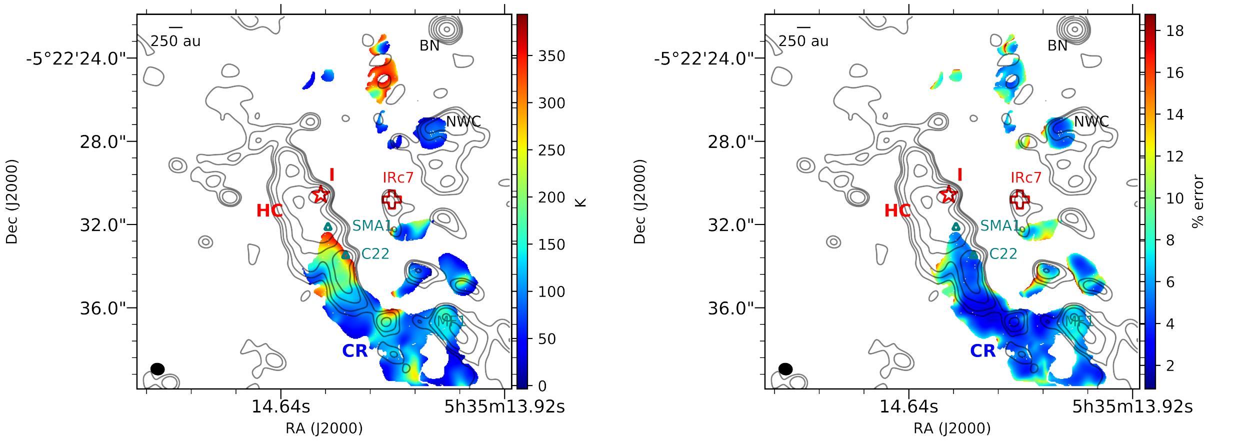

Figure S4: Derived $^{13}CH_3OH$ rotational temperature and percent propagated uncertainty derived by Wilkins et al.[S2] shown by the left and right color maps, respectively. The 2 mm (∼ 150 GHz) continuum emission is shown by the gray contours at $2\sigma_{RMS}, 4\sigma_{RMS}, 8\sigma_{RMS}, 16\sigma_{RMS}, \ldots$ The $0\farcs7$ synthesized beam is shown by the black ellipse in the bottom left corner. Reproduced with permission from Ref. S2.

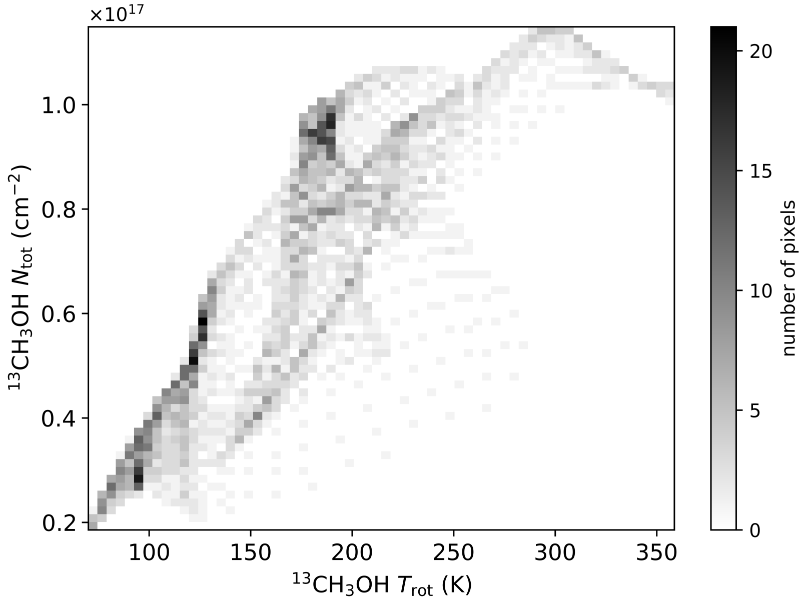

Figure S5: Two-dimensional histogram with 50 points per bin showing the $^{13}CH_3OH$ column density (Figure S3) versus rotational temperature (Figure S4).

S3 Comparison of Derived $[CH_3OD]/[^{12}CH_3OH]$ Ratios

Table S2 shows the corresponding $[CH_3OD]/[^{12}CH_3OH]$ ratios (fourth column) calculated from $[CH_3OD]/[^{13}CH_3OH]$ (between 1.5 and 5.3, see Figure 2b) and different $^{12}C/^{13}C$ ratios reported in the literature. These ranges all roughly encompass the ratio of 0.01-0.06 observed in Orion KL with the IRAM 30m telescope by Mauersberger et al.[S4], but also span higher $[CH_3OD]/[^{12}CH_3OH]$ ratios up to $\sim$0.1. This may suggest CH_3OD abundance enhancements on smaller spatial scales were resolved out in the single-dish data, but that the relative isotopologue abundances presented here are otherwise representative of the flux recovered in single-dish data.

Table S2: $[CH_3OD]/[^{12}CH_3OH]$ (this work) derived from different $^{12}C/^{13}C$ ratios.

$^{12}C/^{13}C$	Observed compound(s)	Location	$[CH_3OD]/[^{12}CH_3OH]$	References
77 ± 7	CO, H_2CO	Local ISM	$0.014 - 0.087$	S5
68 ± 15	CN, CO, H_2CO	Local ISM	$0.015 - 0.104$	S6
63 ± 17	CH_3OH	Orion KL	$0.02 - 0.12$	S7
68.4 ± 10.1	$HCOOCH_3$	Compact Ridge	$0.0156 - 0.1000$	S8
71.4 ± 7.8	$HCOOCH_3$	Hot Core-SW	$0.0153 - 0.0945$	S8

S4 Loss Calculations of CH_3OD by Reactions with OH

We considered the possible loss of gas-phase CH_3OD by the neutral-radical reaction between methanol (CH_3OH) and the hydroxyl radical (OH),

$$CH_3OH + OH \longrightarrow CH_3O + H_2O \tag{1}$$

$$CH_3OH + OH \longrightarrow CH_2OH + H_2O \tag{2}$$

which has been well studied by both theorists and experimentalists.[S9–S12] However, the rate coefficient of this reaction is about two orders of magnitude larger at 60 K, where the methoxy radical (CH_3O, eq 1) is the dominant product, than at 200 K, where the hydroxymethyl radical (CH_2OH, eq 2) dominates.[S9] At 200 K, the combined rate constant for eqs 1 and 2 is reported to be $k = 3.94 \times 10^{-13}$ cm^3 s^{-1} for CH_3OH + OH.[S11] Because of kinetic isotope effects, the actual rate constant is lower (and thus the reaction even slower) for the heavier CH_3OD isotopologue.

We estimate that the reactions in eqs 1 and 2 alone would require $\sim 10^5$ years to deplete the observed enhanced CH_3OD abundances in the Orion KL Hot Core-SW. The combined rate constant k for these two reactions gradually increases with temperature. For simplicity, we make temporal estimates using the value for 200 K, $k = 3.94 \times 10^{-13}$ cm^3 s^{-1}, reported by Gao et al.[S11]

Between 185 and 240 K in Figure 3b, there is a decrease in $N_{\rm tot}(CH_3OD)$ by about 2.5×10^{17} cm^{-2}, or—assuming an object depth of 2.9×10^{16} cm (i.e., if the Hot Core-SW is taken to be $\sim 5''$ across)—$n(CH_3OD) \approx 8.7$ cm^{-3}. This drop comes from an initial abundance of 2.6×10^{17} cm$^{-2} \approx 9.0$ cm^{-3}. The time for this decrease can be approximated as

$$dt = \frac{dn(CH_3OD)}{-k\ n(CH_3OD)} \tag{3}$$

where $n(CH_3OD)$ and $dn(CH_3OD)$ are 9.0 and -8.7 cm^{-3}, respectively. The rate constant k then suggests this reaction would take nearly 2.5×10^{12} s, or $\sim 10^5$ yr, to deplete CH_3OD above 185 K, which is longer than the assumed warm-up time of 10^3 yr that potentially explains the CH_3OD enrichment observed at temperatures of $\sim$100−125 K.

References

(S1) Goldsmith, P. F.; Langer, W. D. Population Diagram Analysis of Molecular Line Emission. *Astrophys. J.* **1999**, *517*, 209–225.

(S2) Wilkins, O. H.; Carroll, P. B.; Blake, G. A. Mapping Physical Parameters in Orion KL at High Spatial Resolution. *Astrophys. J.* **2022**, *924*, 4.

(S3) Favre, C.; Despois, D.; Brouillet, N.; Baudry, A.; Combes, F.; Guélin, M.; Wootten, A.; Wlodarczak, G. $HCOOCH_3$ as a probe of temperature and structure in Orion-KL. *Astron. Astrophys.* **2011**, *532*, A32.

(S4) Mauersberger, R.; Henkel, C.; Jacq, T.; Walmsley, C. M. Deuterated Methanol in Orion. *Astron. Astrophys.* **1988**, *194*, L1–L4.

(S5) Wilson, T. L.; Rood, R. Abundances in the Interstellar Medium. *Annu. Rev. Astron. Astrophys.* **1994**, *32*, 191–226.

(S6) Milam, S. N.; Savage, C.; Brewster, M. A.; Ziurys, L. M.; Wyckoff, S. The $^{12}C/^{13}C$ Isotope Gradient Derived from Millimeter Transitions of CN: The Case for Galactic Chemical Evolution. *Astrophys. J.* **2005**, *634*, 1126–1132.

(S7) Gong, Y.; Henkel, C.; Thorwirth, S.; Spezzano, S.; Menten, K. M.; Walmsley, C. M.; Wyrowski, F.; Mao, R. Q.; Klein, B. A 1.3 cm Line Survey toward Orion KL. *Astron. Astrophys.* **2015**, *581*, A48.

(S8) Favre, C.; Carvajal, M.; Field, D.; Jørgensen, J. K.; Bisschop, S. E.; Brouillet, N.; Despois, D.; Baudry, A.; Kleiner, I.; Bergin, E. A.; Crockett, N. R.; Neill, J. L.; Margulès, L.; Huet, T. R.; Demaison, J. ^{13}C-Methyl Formate: Observations of a Sample of High-mass Star-forming Regions Including orion-KL and Spectroscopic Characterization. *Astrophys. J. Suppl. Ser.* **2014**, *215*, 25.

(S9) Shannon, R. J.; Blitz, M. A.; Goddard, A.; Heard, D. E. Accelerated chemistry in the reaction between the hydroxyl radical and methanol at interstellar temperatures facilitated by tunnelling. *Nature Chemistry* **2013**, *5*, 745–749.

(S10) Acharyya, K.; Herbst, E.; Caravan, R. L.; Shannon, R. J.; Blitz, M. A.; Heard, D. E. The importance of OH radical-neutral low temperature tunnelling reactions in interstellar clouds using a new model. *Molecular Physics* **2015**, *113*, 2243–2254.

(S11) Gao, L. G.; Zheng, J.; Fernández-Ramos, A.; Truhlar, D. G.; Xu, X. Kinetics of the Methanol Reaction with OH at Interstellar, Atmospheric, and Combustion Temperatures. *J. Am. Chem. Soc.* **2018**, *140*, 2906–2918.

(S12) Heard, D. E. Rapid Acceleration of Hydrogen Atom Abstraction Reactions of OH at Very Low Temperatures through Weakly Bound Complexes and Tunneling. *Accounts of Chemical Research* **2018**, *51*, 2620–2627.
